# Impact of HbA1c criterion on the definition of glycemic component of the metabolic syndrome: the China health and nutrition survey 2009

**DOI:** 10.1186/1471-2458-13-1045

**Published:** 2013-11-05

**Authors:** Xingxing Sun, Tingting Du, Rui Huo, Xuefeng Yu, Lixian Xu

**Affiliations:** 1 Department of Anesthesiology, School of Stomatology, Fourth Military Medical University, Xi’an, China; 2 Department of Endocrinology, Tongji Hospital, Tongji Medical College, Huazhong University of Science and Technology, Wuhan, China

**Keywords:** Fasting plasma glucose, HbA1c, Metabolic syndrome, CVD risk factors

## Abstract

**Background:**

In 2009, a unified definition of metabolic syndrome (MetS) was proposed, of which, the glycemic component is defined on the basis of fasting plasma glucose (FPG) level. Recently, the American Diabetes Association (ADA) recommended the use of glycated hemoglobin (HbA1c) as an alternative to FPG to define prediabetes. Hence, we aim to compare the performance of HbA1c and FPG in the definition of glycemic component of the MetS among Chinese adults.

**Methods:**

We conducted a cross-sectional analysis of 7641 Chinese participants aged ≥18 years using data from the China Health and Nutrition Survey 2009. MetS was defined according to the consensus criteria in 2009. We compared the use of HbA1c versus FPG in the definition of the glycemic component of MetS. Increased HbA1c value was defined following the criterion of HbA1c cut-off point of ≥5.7% recommended by the ADA.

**Results:**

Overall, 1136 (14.9%) had MetS according to FPG ≥ 5.6 mmol/l, and 1640 (21.5%) had MetS according to HbA1c ≥ 5.7%. Compared with individuals with FPG-based diagnosis of MetS, individuals with HbA1c-based diagnosis of MetS were older, had higher levels of LDL-C, magnesium, and transferrin, and lower levels of uric acid. Of those found to have MetS according to either FPG or HbA1c (n = 2008), overlap between HbA1c- and FPG-based diagnosis of MetS was limited (n = 768, 38.2%). The overlap index regarding MetS diagnosed by FPG or HbA1c persisted low in each evaluated subgroup (≤ 50.0%).

**Conclusions:**

We note limited overlap and poor agreement between FPG- and HbA1c-based diagnosis of MetS. Screening MetS through introduction of HbA1c in addition to FPG could contribute to identification of more people with MetS.

## Background

The metabolic syndrome (MetS), characterized by abdominal obesity, hypertriglyceridemia, low high-density lipoprotein cholesterol (HDL-C) level, high blood pressure (BP), and increased fasting glucose level, predisposes individuals to a high risk for cardiovascular disease (CVD) and diabetes [1-4]. The MetS is a common disorder in China. According to data from the International Collaborative Study of Cardiovascular Disease in ASIA, 64 (15.1%) million adults aged 35–74 years have the MetS according to the modified Adult Treatment Panel III (ATP III) criteria [5]. The ATP III criteria were slightly modified by lowering the threshold for fasting plasma glucose (FPG) to 5.6 mmol/l in 2004 by the American Heart Association/National Heart, Lung, and Blood Institute (AHA/NHLBI) [6,7] to be consistent with the American Diabetes Association (ADA) criteria for impaired fasting glucose (IFG) [8]. Most recently, several major organizations, led by the International Diabetes Federation (IDF) and AHA/NHLBI, proposed a unified definition of MetS in 2009 [9], while the ADA recommended the use of glycated hemoglobin (HbA1c) as an alternative to IFG to define the category of increased diabetes risk in 2010 [10]. Studies from the USA and Europe showed that HbA1c can be used instead of FPG in identifying individuals with MetS. However, evidence has suggested that HbA1c value might differ according to ethnic origin [11]. Whether HbA1c can be used instead of FPG in the definition of MetS in Chinese population remains unknown. Hence, we took advantage of the large representative sample of Chinese adults who participated in the China Health and Nutrition Survey (CHNS) 2009 to compare the uses of HbA1c ≥ 5.7% or FPG ≥ 5.6 mmol/l in the definition of the glycemic component of MetS. In addition, as prior evidence provided mixed results as to whether the presence of MetS aggravates cardiovascular risk in diabetic patients [12-14], we repeated the analysis in participants without diabetes (FPG ≥7.0 mmol/l or HbA1c ≥6.5%).

## Methods

### Study design

The CHNS, which included a sample representative 56% of the Chinese population, is the only large-scale longitudinal, national survey in China. It was designed to explore how the social and economic transformation of Chinese society is affecting the health and nutritional status of the Chinese population. The CHNS rounds were conducted in 1989, 1991, 1993, 1997, 2000, 2004, 2006, 2009 and 2011. For each round, a stratified multistage, random cluster process was used to draw study sample from nine provinces (Liaoning, Heilongjiang, Jiangsu, Shandong, Henan, Hubei, Hunan, Guangxi and Guizhou) that vary significantly in terms of geography, economic development, and health status. Counties in the nine provinces were stratified by income (low, middle and high) and a weighted sampling scheme was used to select randomly four counties in each province. At last, the CHNS collected health data in 228 communities. Full details of the CHNS have been described elsewhere [15]. Each participant provided a written informed consent and the study was approved by the institutional review committees of the University of North Carolina at Chapel Hill, the National Institute of Nutrition and Food Safety, Chinese Center for Disease Control and Prevention, and the China-Japan Friendship Hospital, Ministry of Health.

### Study population

Since fasting blood samples were initially collected in 2009, this study examined data from the CHNS 2009. The 2009 examination surveyed 10039 participants aged ≥ 18 years. All participants were asked to complete a structured questionnaire which provided information on age, sex, urban/rural settings, educational attainment, histories of current and previous illness, and medical treatment and so on. Participants were included in the present analysis if they were 18 years or older. Exclusion criteria included pregnancy, a self-reported diabetes diagnosis or diabetes medication use, no information on age, body mass index (BMI), HbA1c, or five components of MetS, and anemia (hemoglobin <13 g/dl in men, and <12 g/dl in women). The remaining available 7641 participants with anthropometry and clinical examination information were included in our analysis.

### Anthropometry measurements

Weight, height, waist circumference (WC) and BP were measured following standardized protocols from the World Health Organization (WHO). Weight was measured with the participants wearing light clothing on a calibrated beam balance and height was measured without shoes using a portable SECA stadiometer (Seca North America East, Hanover, MD, USA). Body mass index (BMI) was calculated as weight (in kilograms) divided by the square of height (in meters). WC was measured with an inelastic tape to the nearest 0.1 cm at a midpoint between the bottom of the rib cage and the top of the iliac crest at the end of exhalation. BP was measured by trained examiners using a mercury sphygmomanometer with appropriate cuff at three different consecutive times at 3–5 min intervals on one visit. The three readings were averaged as the BP values in our data analysis. All physical examinations were performed at the same location and followed the same protocol.

### Biochemical measurements

Blood was collected after an overnight fast by venipuncture. Samples for FPG and HbA1c measurements were centrifuged and tested immediately. Serum samples for determinations of CVD risk factors, except for FPG and HbA1c, were then frozen, and stored at −86°C for later laboratory analysis. All blood samples were analyzed in a national central lab in Beijing (medical laboratory accreditation certificate ISO15189:2007). Glucose was measured with a Hitachi 7600 analyzer by GOD-PAP method [Randox Laboratories Ltd, UK]. HbA1c was measured with high performance liquid chromatography (HPLC) system [model HLC-723 G7; Tosoh Corporation, Tokyo, Japan]. Lipids (total cholesterol [TC], triglycerides [TG], low density lipoprotein cholesterol [LDL-C], and HDL-C), serum albumin, uric acid, alanine aminotransferase, magnesium, and hypersensitive-C reactive protein (hs-CRP) were measured using a biochemical autoanalyzer (Hitachi 7600 automated analyzer, Tokyo, Japan). Hs-CRP was determined by the immunoturbidimetric method. Fasting insulin concentration was measured using the radioimmunology assay (Gamma counter XH-6020, China). Homeostasis model assessment of insulin resistance (HOMA2-IR) was calculated using the HOMA2 calculator updated in 2007 because it is more accurate than the original HOMA1 method (based on explicit formulas) [16].

### Definitions

The MetS was defined according to the consensus criteria in 2009 [9]. Persons with MetS are those with the presence of three or more of the following criteria: elevated WC, defined using population- and country-specific cut points (for Chinese, the cut points for WC were ≥ 85 cm in men and ≥ 80 cm in women); TG ≥ 1.7 mmol/l; HDL-C <1.0 mmol/l in men and < 1.3 mmol/l in women; BP ≥ 130/85 mmHg or on antihypertensive drug treatment in a patient with a history of hypertension; or FPG ≥ 5.6 mmol/l. According to 2010 ADA criteria [10], dysglycemia is defined as FPG ≥ 5.6 mmol/l or HbA1c ≥ 5.7%.

### Statistical analysis

All statistical analyses were performed using SPSS software (version 12.0 for windows; SPSS, Chicago, IL, USA). Continuous variables were presented as mean ± SD or medians (25th to 75th percentiles) as appropriate. Categorical variables were presented as numbers and percentages. One-way ANOVA was applied to compare differences in means across groups. Kruskal-Wallis analysis of median test was used followed by the Mann–Whitney *U* test for pairwise comparisons. Chi-square test was performed to assess differences in proportions across groups. Bonferroni correction was applied to adjust P-values for multiple comparisons. Analysis were stratified by sex, age groups (18–44 years, 45–64 years, and ≥ 65 years), urban/rural settings, regions (Southern [Jiangsu, Hubei, Hunan, Guangxi, Guizhou]/Northern[Liaoning, Heilongjiang, Shandong, Henan]), educational attainments (Primary or below, less than high school, high school or above), BMI categories, WC groups, and BP status. The uses of HbA1c ≥ 5.7% or FPG ≥ 5.6 mmol/l in the definition of the glycemic component of MetS were compared. The kappa (ҝ) statistic was calculated to test for an agreement between FPG- and HbA1c-based identification of MetS. Venn diagram was constructed as a visual display of concordance/discordance between FPG- and HbA1c-based identification of MetS. The diagnostic property of HbA1c ≥ 5.7% in identifying MetS was evaluated by receiver operating characteristic (ROC) curve. A two-tailed P value of < 0.05 was considered to be significant.

## Results

### Prevalence of metabolic syndrome

Of the 7641 persons without a history of diabetes, 1136 (14.9%) met MetS using the FPG criterion, and 1640 (21.5%) met MetS using the HbA1c criterion. The proportion of FPG-based diagnosis of MetS remained stable between genders (15.0% in men and 14.8% in women, P = 0.63), while women had a significantly higher proportion of HbA1c-based diagnosis of MetS than men (19.7% in men and 23.0% in women, P < 0.001). The prevalence of FPG- and HbA1c-based diagnosis of MetS increased with age (Table 1). However, the age-related gradient was notably steeper for HbA1c-based definition of MetS than FPG-based definition of MetS. For each age group, the prevalence of HbA1c-based identification of MetS was significantly higher than that of FPG-based definition of MetS. Similar significant trends were noted in urban/rural settings, regions, all education groups, BMI and WC categories, and all BP status.

**Table 1:** Prevalence of metabolic syndrome (MetS) according to fasting plasma glucose or HbA1c or both

	**No MetS**	**FPG-based diagnosis of MetS**	**HbA1c-based diagnosis of MetS**	**MetS by both FPG and HbA1c**	ҝ coefficient^*^
ALL	5633 (73.7)	368 (4.8)	872 (11.4)	768 (10.1)	0.458
Sex					
Men	2731 (75.3)	179 (5.0)	353 (9.7)	361 (10.0)	0.489
Women	2902 (72.3)	189 (4.7)	519 (12.9)	407 (10.1)	0.432
Age, y					
18-44	2434 (86.6)	88 (3.1)	176 (6.3)	113 (4.0)	0.412
45-64	2407 (68.4)	214 (6.1)	475 (13.5)	423 (12.0)	0.431
≥ 65	792 (60.5)	66 (5.0)	221 (16.8)	232 (17.7)	0.474
BMI categories					
< 25 kg/m^2^	4462 (83.1)	189 (3.5)	409 (7.6)	310 (5.8)	0.449
25-30 kg/m^2^	1056 (53.9)	148 (7.6)	395 (20.2)	359 (18.3)	0.376
≥ 30 kg/m^2^	115 (36.8)	31 (9.9)	68 (21.7)	99 (31.6)	0.375
WC categories					
< 85/80 cm	3507 (93.6)	44 (1.2)	119 (3.2)	72 (2.0)	0.448
≥ 85/80 cm	2126 (54.5)	324 (8.3)	753 (19.3)	696 (17.9)	0.371
BP status					
< 120/80 mmHg	2159 (91.5)	46 (2.0)	101 (4.3)	53 (2.2)	0.388
120/80-140/90 mmHg	2532 (75.9)	150 (4.5)	346 (10.4)	307 (9.2)	0.467
≥140/90 mmHg	942 (48.4)	172 (8.8)	425 (21.8)	408 (21.0)	0.349
Settings					
Urban	1785 (72.8)	146 (5.9)	287 (11.7)	236 (9.6)	0.412
Rural	3848 (74.2)	222 (4.3)	585 (11.3)	532 (10.2)	0.478
Regions					
Southern	3438 (78.9)	224 (5.1)	392 (9.0)	304 (7.0)	0.416
Northern	2195 (66.9)	144 (4.4)	480 (14.6)	464 (14.1)	0.481
Education					
Primary or below	2367 (69.5)	171 (5.0)	471 (13.8)	396 (11.7)	0.439
Less than high school	1900 (76.9)	121 (4.9)	232 (9.3)	220 (8.9)	0.472
High school or above	1366 (77.5)	76 (4.3)	169 (9.6)	152 (8.6)	0.474

Data are numbers (percentages).

^*^An agreement between FPG- and HbA1c- based identification of MetS.

FPG, fasting plasma glucose; BMI, body mass index; WC, waist circumference; BP, blood pressure.

### Overlap between FPG- and HbA1c-based definition of metabolic syndrome

**Figure 1:**
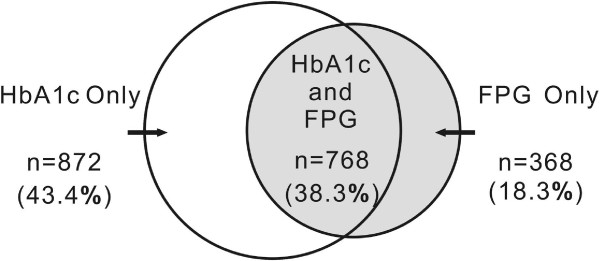
Venn diagram for individuals meeting FPG- and HbA1c-based diagnosis of metabolic syndrome: the CHNS 2009.

Of those found to have MetS by either FPG or HbA1c (n = 2008, 26.3%), overlap between HbA1c- and FPG-based diagnosis of MetS was limited (n = 768, 38.2%) (Figure 1). The ҝ coefficient of the FPG criterion with HbA1c criterion for the diagnosis of MetS was 0.458. Using FPG-based diagnosis of MetS as the reference standard, the HbA1c cut-point of 5.7% in identifying subjects with MetS demonstrated a sensitivity of 60.3%, specificity of 76.2%, positive predictive value of 30.6%, and negative predictive value of 91.7%. ROC curve analysis with HbA1c as a continuous variable yielded an area under the curve of 0.74 (P < 0.01). The overlap index regarding MetS diagnosed by FPG and HbA1c was low irrespective of sex, age, urban/rural setting, region, educational attainment, BMI, WC and BP status (Table 2). The magnitude of overlap between the two criteria slightly increased with increasing age, worsening BMI, WC, and BP.

**Table 2:** Proportions of metabolic syndrome (MetS) by fasting plasma glucose only, HbA1c only, and by both criteria

	**MetS by either FPG or HbA1c**	**FPG-based diagnosis of MetS**	**HbA1c-based diagnosis of MetS**	MetS by both FPG and HbA1c (overlap^*^)
Sex				
Men	893	179 (20.0)	353 (39.5)	361 (40.4)
Women	1115	189 (17.0)	519 (46.5)	407 (36.5)
Age, y				
18-44	377	88 (23.3)	176 (46.7)	113 (30.0)
45-64	1112	214 (19.2)	475 (42.7)	423 (38.0)
≥ 65	519	66 (12.7)	221 (42.6)	232 (44.7)
BMI categories				
< 25 kg/m^2^	908	189 (20.8)	409 (45.0)	310 (34.1)
25-30 kg/m^2^	902	148 (16.4)	395 (43.8)	359 (39.8)
≥ 30 kg/m^2^	198	31 (15.7)	68 (34.3)	99 (50.0)
WC categories				
< 85/80 cm	235	44 (18.7)	119 (50.6)	72 (30.6)
≥ 85/80 cm	1773	324 (18.3)	753 (42.5)	696 (39.3)
BP status				
< 120/80 mmHg	200	46 (23.0)	101 (50.5)	53 (26.5)
120/80-140/90 mmHg	803	150 (18.7)	346 (43.1)	307 (38.2)
≥140/90 mmHg	1005	172 (17.1)	425 (42.3)	408 (40.6)
Settings				
Urban	669	146 (21.8)	287 (42.9)	236 (35.3)
Rural	1339	222 (16.6)	585 (43.7)	532 (39.7)
Regions				
Southern	920	224 (24.3)	392 (42.6)	304 (33.0)
Northern	1088	144 (13.2)	480 (44.1)	464 (42.6)
Education				
Primary or below	1038	171 (16.5)	471 (45.4)	396 (38.2)
Less than high school	573	121 (21.1)	232 (40.5)	220 (38.4)
High school or above	397	76 (19.1)	169 (42.6)	152 (38.3)

^*^Proportions of overlap between HbA1c- and FPG-based diagnosis of MetS are calculated as the number of MetS by both FPG and HbA1c divided by the number of MetS based on either FPG or HbA1c.

FPG, fasting plasma glucose; BMI, body mass index; WC, waist circumference; BP, blood pressure.

Similar to the overall cohort, in the non-diabetes group, overlap between HbA1c- and FPG-based diagnosis of MetS was even more limited (5.9%). The ҝ coefficient of the FPG criterion with HbA1c criterion for the diagnosis of MetS was 0.322.

### Characteristics of individuals diagnosed with metabolic syndrome by each measure

As expected, the worst CVD risk profile was found in individuals with MetS based upon both FPG and HbA1c and the most favorable cardiovascular profile was found in individuals without MetS (Table 3). The characteristics differed for individuals with FPG-based diagnosis of MetS compared with participants with HbA1c-based diagnosis of MetS. Specifically, participants with HbA1c-based diagnosis of MetS were older, had higher levels of HbA1c, LDL-C, magnesium, and transferrin, and lower levels of FPG, and uric acid. Results were largely replicated when the same analysis were conducted in the non-diabetes group (Additional file 1).

**Table 3:** Characteristics of participants stratified by diagnosis of metabolic syndrome (MetS) according to fasting plasma glucose or HbA1c or both

	**No MetS**	**FPG-based diagnosis of MetS**	**HbA1c-based diagnosis of MetS**	**MetS by both FPG and HbA1c**
Female (%)	51.5	51.4	59.5	53.0
Age (years)^§^	48.2 ± 15.0^*† ‡^	53.6 ± 12.2^*^	56.1 ± 13.2^*^	58.2 ± 12.1
Body mass index (kg/m^2^)^§^	22.6 ± 3.1^*† ‡^	25.3 ± 3.1^*^	25.4 ± 3.1^*^	26.1 ± 3.4
Waist circumference (cm)^§^	79.8 ± 9.4^*† ‡^	89.1 ± 8.0^*^	89.3 ± 8.4^*^	91.5 ± 8.7
Systolic blood pressure (mmHg)	120.0 (110.0-128.7)^*† ‡^	130.0 (120.0-142.3)^*^	131.0 (120.0-144.7)^*^	136.0 (123.3-149.3)
Diastolic blood pressure (mmHg)	79.3 (70.7-83.3)^*† ‡^	85.0 (80.0-90.7)	86.0 (80.0-91.0)	86.7 (80.0-91.8)
Fasting plasma glucose (mmol/l)	4.9 (4.6-5.3)^*† ‡^	5.9 (5.7-6.3)	5.0 (4.7-5.3)^*†^	6.4 (5.9-7.5)
HbA1c (%)	5.4 (5.1-5.6)^*‡^	5.4 (5.2-5.5)^*^	5.9 (5.8-6.1)^*†^	6.2 (5.9-6.8)
Total cholesterol (mmol/l)	4.7 (4.1-5.3)^*‡^	4.9 (4.3-5.7)^*^	5.0 (4.5-5.7)^*^	5.3 (4.7-6.0)
Triglycerides (mmol/l)	1.1 (0.8-1.5)^*† ‡^	2.0 (1.3-2.7)	1.8 (1.2-2.5)	1.9 (1.3-2.8)
LDL cholesterol (mmol/l)	2.8 (2.3-3.4)^*† ‡^	3.0 (2.3-3.5)^*^	3.1 (2.5-3.8)^†^	3.3 (2.6-3.9)
HDL cholesterol (mmol/l)	1.4 (1.2-1.7)^*† ‡^	1.2 (1.0-1.5)	1.2 (1.1-1.5)	1.2 (1.0-1.4)
Uric acid (mmol/l)	286.0 (233.0-349.0)^*† ‡^	336.5 (281.5-411.0)	318.0 (256.0-377.0))^*†^	328.0 (267.0-393.0)
HOMA2-IR	1.3 (0.9-1.8)^*† ‡^	1.9 (1.4-2.9)^*^	1.6 (1.2-2.2)^*^	2.2 (1.5-3.5)
Hemoglobin (g/dl)	141.0 (130.0-153.0)^*†^	145.0 (133.0-157.0)	142.0 (131.0-154.0)^*^	146.0 (135.0-159.0)
Albumin (g/dl)	47.3 (45.3-49.5)	47.5 (45.5-49.7)	47.3 (45.3-49.3)	47.8 (45.7-49.8)
Alanine aminotransferase (UI/L)	17.0 (13.0-25.0)^*† ‡^	21.0 (14.0-31.5)^*^	21.0 (15.0-29.0)^*^	23.0 (17.0-32.5)
Estimated glomerular filtration rate (ml/min per 1.73 m^2^)	76.7 (68.2-86.6)^*†^	73.7 (65.0-82.8)	74.4 (66.2-83.3)	72.8 (63.4-82.2)
C-reactive protein (mg/l)	1.0 (0.0-2.0)^*‡^	1.0 (1.0-3.0)^*^	2.0 (1.0-3.0)	2.0 (1.0-4.0)
Serum magnesium	0.9 (0.9-1.0)^*‡^	0.9 (0.9-1.0)^*^	1.0 (0.9-1.0)^†^	1.0 (0.9-1.0)
Transferrin (ng/ml)	2.8 (2.5-3.1)^*‡^	2.8 (2.5-3.1)^*^	2.9 (2.6-3.3)^†^	3.0 (2.6-3.3)
Soluble Transferrin receptor (ng/ml)	1.3 (1.1-1.6)	1.3 (1.1-1.7)	1.3 (1.0-1.6)	1.3 (1.1-1.6)
White blood cell count (×10^9^/ml)	6.0 (5.0-7.1)^*‡^	6.1 (5.1-7.3)^*^	6.2 (5.3-7.5)^*^	6.5 (5.6-7.7)

Data are medians (25th to 75th percentiles) or percent, except variables denoted by ^§^, which are means ± SD.

^*^P < 0.001 compared with individuals with MetS according to both FPG and HbA1c.

^†^P < 0.001 compared with individuals with MetS according to FPG only.

^‡^P < 0.001 compared with individuals with MetS according to HbA1c only.

## Discussion

In the present study, we compared HbA1c and FPG in the definition of glycemic component of MetS. We saw low magnitude of overlap and poor agreement between FPG- and HbA1c-based identification of MetS. Similar significant trends were noted in each evaluated subgroup. The prevalence of HbA1c-based diagnosis of MetS was significantly higher than FPG-based diagnosis of MetS. Screening MetS by introduction of the new HbA1c criterion in addition to assessment of the FPG could contribute to identification of more people with MetS.

Our results are in line with the study from an European country with respect to a higher prevalence of MetS measured by HbA1c than by FPG [17]. Evidence has suggested that there were a large proportion of Chinese with isolated postprandial hyperglycemia [18]. Although postprandial glucose were not available in the present study, it is possible that even with normal fasting glucose, individuals may have postprandial hyperglycemia. HbA1c represents chronic exposure to basal and postprandial hyperglycemia. Hence, HbA1c can identify more individuals with MetS than FPG.

In the present CHNS cohort, HbA1c-based diagnosis of MetS illustrated limited overlap with FPG-based diagnosis of MetS. This deviates from the findings of the cohort studies from United States and European countries in which a good agreement between HbA1c- and FPG-based identification of MetS was observed [17,19]. Hence, a discrepancy in the degree of overlap might be more likely to happen in Chinese than in US and European adults. The inconsistency between the cited studies and our present study may be attributed to the discrepancies in ethnicity, socio-demographic or personal characteristics, prevalence of CVD risk factors such as hypertension, obesity, hypertriglyceridemia, and lifestyle differences. Another possible explanation for the inconsistency might be due to the fact that FPG differentially correlated with HbA1c among different ethnic populations as several recent reports have compared the relationship of HbA1c to FPG in various populations and have found significant discordance [11,20]. HbA1c correlates better with insulin resistance than FPG [21]. To date, differences in the relation of FPG and HbA1c to insulin resistance and insulin secretion have been little described in Chinese population. One study conducted in Japanese population indicated that high normal HbA1c levels (5.4–5.8%) were associated with impaired insulin secretion without marked insulin resistance and elevated HbA1c levels (≥5.9%) were associated with substantial impairment in insulin secretion, insulin sensitivity and β-cell dysfunction [21], while one Chinese population based study suggested that increased FPG is predominately induced by the decline in insulin sensitivity and insulin secretion, with the insulin secretion more pronounced [22]. Taken together, it is possible that how well HbA1c performs as compared with FPG for the identification of MetS will depend on the target population.

The limited overlap between HbA1c- and FPG-based diagnosis of MetS highlights different facets of glucose metabolism and multifactorial pathophysiologic mechanisms for glucose dysfunction [23-25]. In contrast to daily pre-prandial glucose snapshot offered by FPG, HbA1c captures chronic hyperglycemia, including postprandial glucose spikes. Increased FPG is predominately induced by liver insulin resistance and a defect in the early phase of insulin secretion [23,25], whereas increased HbA1c is dominated by a combination of hepatic insulin resistance, muscle insulin resistance and impaired insulin secretion [24,25]. Although the explanations for the significantly higher proportion of HbA1c-based diagnosis of MetS in women compared with men observed in the present study remain to be elucidated, it is probably related to gender differences in glucose absorption patterns [26]. The difference in gender-specific glucose absorption pattern is a consequence of the relatively higher dose of glucose given to women compared with men when seen in relation to their body size [27,28]. Several studies have shown that men in general have a higher prevalence of isolated impaired fasting glucose than do women [27,28]. In contrast, women often exhibit a higher prevalence of isolated impaired glucose tolerance [27,28]. Monnier et al. found that the relative contribution of postprandial hyperglycemia accounted for ~70% of overall glycemic exposure in patients with HbA1c <7.3%, while the relative contribution of FPG increased gradually with increasing levels of HbA1c [29]. In the present study, we noted that only 8 (0.9%) individuals had HbA1c ≥7.3% among the 872 individuals with HbA1c-based definition of MetS (data not shown), indicating a greater contribution of postprandial versus FPG to HbA1c levels. Hence, the HbA1c-based definition of MetS reflects more of the postprandial glucose-based diagnosis of MetS. Taken together, it is possible that women carry a higher prevalence of HbA1c-based diagnosis of MetS than do men. A greater contribution of postprandial versus FPG to HbA1c levels may also in part explain the limited overlap between FPG and HbA1c-based definition of MetS.

Subgroup differences were noted in the present study, with a slightly higher magnitude of overlap between FPG and HbA1c-based definition of MetS among persons with older age, elevated BMI, WC, and BP. One possible explanation for this relatively low discrepancy in older age might be the fact that the prevalence of MetS was more frequent among older persons [5,30]. The relatively low discrepancy in worsening BMI, WC, and BP status might be attributed to the fact that obesity, enlarged waist, and hypertension accompany the insulin-resistant state that underlies the MetS.

In this study, individuals with FPG-based identification of MetS had different characteristics compared with individuals with HbA1c-based identification of MetS. The different characteristics might affect progression to CVD mortality and morbidity within each case of discordantly diagnosed MetS. Most components of the MetS are required to be conducted in the fasting state, although an overnight fast is not required for HbA1c measurement, it is possible that clinicians can have access to both FPG and HbA1c in most of their patients. In this context, and based on our data, the MetS rate may increase by approximately 70% if both FPG and HbA1c are measured. Although there is controversy whether the diagnosis of MetS conveys additional cardiovascular risk in subjects with diabetes [12-14], a recent meta-analysis evidenced that in the absence of type 2 diabetes mellitus, the MetS was associated with an increased risk of cardiovascular mortality (RR: 1.75; 95% CI: 1.19 to 2.58) [3]. Our present study showed that in participants without diabetes, even more limited overlap between HbA1c- and FPG-based diagnosis of MetS was observed. The identification of MetS is of clinical importance because appropriate interventions can clearly prevent or delay the CVD mortality and morbidity, especially in subjects without diabetes. Although evidence suggested that there was no linear relationship between FPG and CVD mortality and morbidity [31], the risk for diabetes and CVD mortality and morbidity increased gradually with rising FPG and HbA1c [32,33]. Two prospective studies demonstrated that the combined use of FPG and HbA1c were more predictive of the risk of future diabetes [34,35]. Our finding that individuals with Mets based on both FPG and HbA1c were older, more obese, hypertensive, dyslipidemic, and insulin resistant and had higher CRP compared with individuals with Mets based on FPG alone or HbA1c alone further supported the notion. Therefore, individuals with Mets based on both measure might allow for assessment of a substantially increased risk of CVD mortality and morbidity. We believe that introduction of HbA1c into FPG is appropriate for detection of MetS, and will contribute to cover a larger number of people being submitted to aggressive drug regimens to prevent future risk for diabetes and CVD mortality and morbidity. Further large-scale longitudinal studies are, however, required to address this issue.

Our study has several strengths including a well-established cohort of nationally representative sample of the Chinese adult population, a vigorous quality assurance program, the same strict methodology used to ensure the quality of the data collection over the entire study period, and the centralization of laboratory measurements. We also benefited from the CHNS 2009′s availability of broad range of demographic, clinical, and biomarker data collected by trained staff. Nevertheless, the current study was subjected to several limitations. First, we got availability of only single measures of all biochemical variables, a common limitation to most large epidemiologic studies. Second, since the current study is a cross-sectional design, we cannot explore whether diagnosis of MetS based on different measures have the same predictive capacity in the risk of future diabetes and CVD mortality and morbidity. Third, estimates across subgroups should also be interpreted with caution because of limited sample size.

## Conclusion

In summary, limited overlap and poor agreement are demonstrated between the FPG- and HbA1c-based diagnosis of MetS. Screening MetS through introduction of HbA1c in addition to FPG could contribute to identification of more people with MetS who would otherwise have been missed.

## Abbreviations

MetS: Metabolic syndrome; HbA1c: Glycated hemoglobin; FPG: Fasting plasma glucose; HDL-C: High-density lipoprotein cholesterol; BP: Blood pressure; CVD: Cardiovascular disease; CHNS: China Health and Nutrition Survey; WC: Waist circumference; BMI: Body mass index; TC: Total cholesterol; TG: Triglycerides; LDL-C: Low density lipoprotein cholesterol; hs-CRP: Hypersensitive-C reactive protein; HOMA-IR: Homeostasis model assessment of insulin resistance; HOMA-β: Homeostasis model assessment of beta-cell function.

## Competing interests

No potential conflicts of interest relevant to this article were reported. The authors declare that they have no competing interests.

## Authors’ contributions

XXS and TTD conceived the study, completed all statistical analyses, and drafted the manuscript. LXX, XFY and RH contributed to the discussion. LXX and XFY revised the manuscript. All authors have read and approved the final manuscript.

## Pre-publication history

The pre-publication history for this paper can be accessed here:

http://www.biomedcentral.com/1471-2458/13/1045/prepub

## Supplementary Material

Additional file 1 Characteristics of CHNS 2009 participants stratified by diagnosis of MetS according to fasting plasma glucose or HbA1c or both in participants without diabetes. Click here for file

## References

[B1] Wilson PW D’Agostino RB Parise H Sullivan L Meigs JB Metabolic syndrome as a precursor of cardiovascular disease and type 2 diabetes mellitus Circulation 2005 13 3066 3072 16275870 10.1161/CIRCULATIONAHA.105.539528

[B2] Lakka HM Laaksonen DE Lakka TA Niskanen LK Kumpusalo E Tuomilehto J Salonen JT The metabolic syndrome and total and cardiovascular disease mortality in middle-aged men JAMA 2002 13 2709 2716 12460094 10.1001/jama.288.21.2709

[B3] Mottillo S Filion KB Genest J Joseph L Pilote L Poirier P Rinfret S Schiffrin EL Eisenberg MJ The metabolic syndrome and cardiovascular risk a systematic review and meta-analysis J Am Coll Cardiol 2010 13 1113 1132 20863953 10.1016/j.jacc.2010.05.034

[B4] Wannamethee SG Shaper AG Lennon L Morris RW Metabolic syndrome vs Framingham Risk Score for prediction of coronary heart disease, stroke, and type 2 diabetes mellitus Arch Int Med 2005 13 2644 2650 16344423 10.1001/archinte.165.22.2644

[B5] Gu D Reynolds K Wu X Chen J Duan X Reynolds RF Whelton PK He J Inter ACG Prevalence of the metabolic syndrome and overweight among adults in China Lancet 2005 13 1398 1405 15836888 10.1016/S0140-6736(05)66375-1

[B6] Grundy SM Brewer HB Jr Cleeman JI Smith SC Jr Lenfant C Definition of metabolic syndrome: Report of the National Heart, Lung, and Blood Institute/American Heart Association conference on scientific issues related to definition Circulation 2004 13 433 438 14744958 10.1161/01.CIR.0000111245.75752.C6

[B7] Grundy SM Cleeman JI Daniels SR Donato KA Eckel RH Franklin BA Gordon DJ Krauss RM Savage PJ Smith SC Jr Diagnosis and management of the metabolic syndrome: an American Heart Association/National Heart, Lung, and Blood Institute Scientific Statement Circulation 2005 13 2735 2752 16157765 10.1161/CIRCULATIONAHA.105.169404

[B8] Genuth S Alberti KG Bennett P Buse J Defronzo R Kahn R Kitzmiller J Knowler WC Lebovitz H Lernmark A Follow-up report on the diagnosis of diabetes mellitus Diabetes Care 2003 13 3160 3167 14578255 10.2337/diacare.26.11.3160

[B9] Alberti KG Eckel RH Grundy SM Zimmet PZ Cleeman JI Donato KA Fruchart JC James WP Loria CM Smith SC Jr Harmonizing the metabolic syndrome: a joint interim statement of the International Diabetes Federation Task Force on Epidemiology and Prevention; National Heart, Lung, and Blood Institute; American Heart Association; World Heart Federation; International Atherosclerosis Society; and International Association for the Study of Obesity Circulation 2009 13 1640 1645 19805654 10.1161/CIRCULATIONAHA.109.192644

[B10] American Diabetes Association Diabetes Care 2010 13 Suppl 1 S62 S69 10.2337/dc10-S062 PMC2797383 20042775

[B11] Herman WH Ma Y Uwaifo G Haffner S Kahn SE Horton ES Lachin JM Montez MG Brenneman T Barrett-Connor E Differences in A1C by race and ethnicity among patients with impaired glucose tolerance in the Diabetes Prevention Program Diabetes Care 2007 13 2453 2457 17536077 10.2337/dc06-2003 PMC2373980

[B12] Hadaegh F Shafiee G Ghasemi A Sarbakhsh P Azizi F Impact of metabolic syndrome, diabetes and prediabetes on cardiovascular events: Tehran lipid and glucose study Diab Res Clin Pract 2010 13 342 347 10.1016/j.diabres.2009.11.010 20004035

[B13] Bonora E Targher G Formentini G Calcaterra F Lombardi S Marini F Zenari L Saggiani F Poli M Perbellini S The metabolic syndrome is an independent predictor of cardiovascular disease in type 2 diabetic subjects. Prospective data from the Verona diabetes complications study Diab Med 2004 13 52 58 10.1046/j.1464-5491.2003.01068.x 14706054

[B14] Church TS Thompson AM Katzmarzyk PT Sui X Johannsen N Earnest CP Blair SN Metabolic syndrome and diabetes, alone and in combination, as predictors of cardiovascular disease mortality among men Diabetes Care 2009 13 1289 1294 19366967 10.2337/dc08-1871 PMC2699717

[B15] Popkin BM Du S Zhai F Zhang B Cohort Profile: The China Health and Nutrition Survey–monitoring and understanding socio-economic and health change in China, 1989–2011 Int J Epidemiol 2010 13 1435 1440 19887509 10.1093/ije/dyp322 PMC2992625

[B16] Wallace TM Levy JC Matthews DR Use and abuse of HOMA modeling Diabetes Care 2004 13 1487 1495 15161807 10.2337/diacare.27.6.1487

[B17] Succurro E Marini MA Arturi F Grembiale A Fiorentino TV Andreozzi F Sciacqua A Lauro R Hribal ML Perticone F Usefulness of hemoglobin A1c as a criterion to define the metabolic syndrome in a cohort of italian nondiabetic white subjects Am J Cardiol 2011 13 1650 1655 21420057 10.1016/j.amjcard.2011.01.055

[B18] Yang W Lu J Weng J Jia W Ji L Xiao J Shan Z Liu J Tian H Ji Q Prevalence of diabetes among men and women in China N Engl J Med 2010 13 1090 1101 20335585 10.1056/NEJMoa0908292

[B19] Ong KL Tso AW Lam KS Cherny SS Sham PC Cheung BM Using glycosylated hemoglobin to define the metabolic syndrome in United States adults Diabetes Care 2010 13 1856 1858 20504895 10.2337/dc10-0190 PMC2909078

[B20] Ziemer DC Kolm P Weintraub WS Vaccarino V Rhee MK Twombly JG Narayan KM Koch DD Phillips LS Glucose-independent, black-white differences in hemoglobin A1c levels: a cross-sectional analysis of 2 studies Ann Intern Med 2010 13 770 777 20547905 10.7326/0003-4819-152-12-201006150-00004

[B21] Heianza Y Arase Y Fujihara K Tsuji H Saito K Hsieh SD Kodama S Shimano H Yamada N Hara S High normal HbA(1c) levels were associated with impaired insulin secretion without escalating insulin resistance in Japanese individuals: the Toranomon Hospital Health Management Center Study 8 (TOPICS 8) Diab Med 2012 13 1285 1290 10.1111/j.1464-5491.2012.03667.x 22486679

[B22] Bi Y Zeng L Zhu D Yan J Zhang Y Tong G Mu P Shen S Hu Y Yu Q Association of beta-cell function and insulin sensitivity with fasting and 2-h plasma glucose in a large Chinese population Diab Obes Metab 2012 13 174 180 10.1111/j.1463-1326.2011.01504.x 21951345

[B23] Nathan DM Davidson MB DeFronzo RA Heine RJ Henry RR Pratley R Zinman B American Diabetes A Impaired fasting glucose and impaired glucose tolerance: implications for care Diabetes Care 2007 13 753 759 17327355 10.2337/dc07-9920

[B24] Goldstein DE Little RR Lorenz RA Malone JI Nathan D Peterson CM Sacks DB Tests of glycemia in diabetes Diabetes Care 2004 13 1761 1773 15220264 10.2337/diacare.27.7.1761

[B25] Abdul-Ghani MA Jenkinson CP Richardson DK Tripathy D DeFronzo RA Insulin secretion and action in subjects with impaired fasting glucose and impaired glucose tolerance: results from the Veterans Administration Genetic Epidemiology Study Diabetes 2006 13 1430 1435 16644701 10.2337/db05-1200

[B26] Faerch K Pacini G Nolan JJ Hansen T Tura A Vistisen D Impact of glucose tolerance status, Sex, and body size on glucose absorption patterns during OGTTs Diabetes care 2013 13 3691 3697 24062321 10.2337/dc13-0592 PMC3816886

[B27] Faerch K Borch-Johnsen K Vaag A Jorgensen T Witte DR Sex differences in glucose levels: a consequence of physiology or methodological convenience? The Inter99 study Diabetologia 2010 13 858 865 20182862 10.1007/s00125-010-1673-4

[B28] Williams JW Zimmet PZ Shaw JE de Courten MP Cameron AJ Chitson P Tuomilehto J Alberti KG Gender differences in the prevalence of impaired fasting glycaemia and impaired glucose tolerance in Mauritius. Does sex matter? Diab Med 2003 13 915 920 10.1046/j.1464-5491.2003.01059.x 14632717

[B29] Monnier L Lapinski H Colette C Contributions of fasting and postprandial plasma glucose increments to the overall diurnal hyperglycemia of type 2 diabetic patients: variations with increasing levels of HbA(1c) Diabetes Care 2003 13 881 885 12610053 10.2337/diacare.26.3.881

[B30] Ford ES Giles WH Dietz WH Prevalence of the metabolic syndrome among US adults: findings from the third National Health and Nutrition Examination Survey JAMA 2002 13 356 359 11790215 10.1001/jama.287.3.356

[B31] Sarwar N Gao P Seshasai SR Gobin R Kaptoge S Di Angelantonio E Ingelsson E Lawlor DA Selvin E Emerging Risk Factors C Diabetes mellitus, fasting blood glucose concentration, and risk of vascular disease: a collaborative meta-analysis of 102 prospective studies Lancet 2010 13 2215 2222 20609967 10.1016/S0140-6736(10)60484-9 PMC2904878

[B32] Droumaguet C Balkau B Simon D Caces E Tichet J Charles MA Eschwege E Use of HbA1c in predicting progression to diabetes in French men and women: data from an Epidemiological Study on the Insulin Resistance Syndrome (DESIR) Diabetes Care 2006 13 1619 1625 16801588 10.2337/dc05-2525

[B33] Selvin E Steffes MW Zhu H Matsushita K Wagenknecht L Pankow J Coresh J Brancati FL Glycated hemoglobin, diabetes, and cardiovascular risk in nondiabetic adults N Engl J Med 2010 13 800 811 20200384 10.1056/NEJMoa0908359 PMC2872990

[B34] Heianza Y Hara S Arase Y Saito K Fujiwara K Tsuji H Kodama S Hsieh SD Mori Y Shimano H HbA1c 5.7-6.4% and impaired fasting plasma glucose for diagnosis of prediabetes and risk of progression to diabetes in Japan (TOPICS 3): a longitudinal cohort study Lancet 2011 13 147 155 21705064 10.1016/S0140-6736(11)60472-8

[B35] Selvin E Steffes MW Gregg E Brancati FL Coresh J Performance of A1C for the classification and prediction of diabetes Diabetes Care 2011 13 84 89 20855549 10.2337/dc10-1235 PMC3005486

