# Supplementary material for: Impact of HbA1c criterion on the definition of glycemic component of the metabolic syndrome: the China health and nutrition survey 2009
Source: BMC Public Health. 2013 Nov 5;13:1045. doi: 10.1186/1471-2458-13-1045 (PMC3924337; doi:10.1186/1471-2458-13-1045)
Supplement: Additional file 1 — Characteristics of CHNS 2009 participants stratified by diagnosis of MetS according to fasting plasma glucose or HbA1c or both in participants without diabetes. [file 1471-2458-13-1045-S1.docx]

# Additional file 1

# Characteristics of CHNS 2009 participants stratiﬁed by diagnosis of MetS according to fasting plasma glucose or HbA1c or both in participants without diabetes

|  | No MetS | FPG-based diagnosis of MetS | HbA1c-based diagnosis of MetS | MetS by both FPG and HbA1c |
| --- | --- | --- | --- | --- |
| Female (%) | 51.7 | 52.8 | 59.6 | 53.5 |
| Age (years) ^§^ | 47.9±14.9^*† ‡^ | 53.6±12.2^*^ | 55.9±13.2^*^ | 57.6±11.9 |
| Body mass index (kg/m^2^) ^§^ | 22.6±3.1^*† ‡^ | 25.4±3.1^*^ | 25.4±3.1^*^ | 26.1±3.2 |
| Waist circumference (cm) ^§^ | 79.8±9.4^*† ‡^ | 89.3±7.9^*^ | 89.1±8.4^*^ | 90.9±8.3 |
| Systolic blood pressure (mmHg) | 120.0 (110.0-128.7) ^*† ‡^ | 130.3 (120.0-142.0) ^*^ | 130.7 (120.0-144.7) ^*^ | 135.3 (122.0-147.3) |
| Diastolic blood pressure (mmHg) | 79.3 (70.7-83.3) ^*† ‡^ | 85.3 (80.0-90.7) | 86.0 (80.0-91.0) | 86.7 (80.0-92.0) |
| Fasting plasma glucose (mmol/l) | 4.9 (4.6-5.3) ^*† ‡^ | 5.9 (5.7-6.1) | 5.0 (4.7-5.3) ^*†^ | 6.0 (5.8-6.3) |
| HbA1c (%) | 5.4 (5.1-5.6) ^*‡^ | 5.4 (5.2-5.5) ^*^ | 5.9 (5.8-6.0) ^*†^ | 6.0 (5.8-6.2) |
| Total cholesterol (mmol/l) | 4.6 (4.1-5.3) ^*‡^ | 5.0 (4.3-5.7) ^*^ | 5.0 (4.5-5.7) ^*^ | 5.3 (4.7-6.0) |
| Triglycerides (mmol/l) | 1.1 (0.8-1.5) ^*† ‡^ | 1.9 (1.3-2.7) | 1.8 (1.2-2.4) | 1.8 (1.3-2.5) |
| LDL cholesterol (mmol/l) | 2.8 (2.3-3.4) ^*† ‡^ | 3.0 (2.4-3.5) ^*^ | 3.1 (2.5-3.7) ^*^ | 3.4 (2.7-3.9) |
| HDL cholesterol (mmol/l) | 1.4 (1.2-1.7) ^*† ‡^ | 1.2 (1.0-1.4) | 1.2 (1.1-1.5) | 1.2 (1.1-1.5) |
| Uric acid (mmol/l) | 286.0 (233.0-349.0) ^*† ‡^ | 338.5 (282.0-414.0) | 318.0 (255.0-376.0) ^*†^ | 326.0 (266.0-383.0) |
| HOMA2-IR | 1.3 (0.9-1.8) ^*† ‡^ | 1.8 (1.3-2.7) | 1.6 (1.2-2.2) ^*^ | 2.0 (1.4-3.2) |
| Hemoglobin (g/dl) | 141.0 (130.0-153.0) ^*†^ | 145.0 (133.0-156.0) | 142.0 (131.0-154.0) | 145.0 (134.0-157.0) |
| Alanine aminotransferase (UI/L) | 17.0 (13.0-25.0) ^*† ‡^ | 20.5 (14.0-31.0) ^*^ | 21.0 (15.0-29.0) ^*^ | 22.0 (16.0-30.0) |
| Estimated glomerular ﬁltration rate (ml/min per 1.73 m ^2^) | 76.8 (68.4-86.7) ^*†^ | 73.5 (65.0-83.0) | 74.5 (66.1-83.4) | 73.2 (65.0-82.1) |
| C-reactive protein (mg/l) | 1.0 (0.1-2.0) ^*‡^ | 1.0 (1.0-3.0) ^*^ | 1.0 (1.0-3.0) | 2.0 (1.0-3.0) |
| Serum magnesium | 0.9 (0.9-1.0) ^*‡^ | 0.9 (0.9-1.0) ^*^ | 1.0 (0.9-1.0) | 1.0 (0.9-1.0) |
| Transferrin (ng/ml) | 2.8 (2.5-3.1) ^*^ | 2.8 (2.5-3.1) | 2.9 (2.6-3.2) | 2.9 (2.6-3.3) |
| White blood cell count (×10^9^/ml) | 6.0 (5.0-7.1) ^*‡^ | 6.1 (5.1-7.2) ^*^ | 6.2 (5.3-7.4) ^*^ | 6.4 (5.5-7.4) |

Data are medians (25th to 75th percentiles) or percent, except variables denoted by ^§^, which are means ± SD.

^*^P < 0.001 compared with individuals with MetS according to both FPG and HbA1c;

^†^ P < 0.001 compared with individuals with MetS according to FPG only;

^‡^ P < 0.001 compared with individuals with MetS according to HbA1c only.
